# Genomic mapping identifies two genetic variants in the *MC1R* gene for coat colour variation in Chinese Tan sheep

**DOI:** 10.1371/journal.pone.0235426

**Published:** 2020-08-20

**Authors:** Gebremedhin Gebreselassie, Benmeng Liang, Haile Berihulay, Rabul Islam, Adam Abied, Lin Jiang, Zhengwei Zhao, Yuehui Ma

**Affiliations:** 1 Key Laboratory of Animal (Poultry) Genetics Breeding and Reproduction, Ministry of Agriculture, Institute of Animal Science, Chinese Academy of Agricultural Sciences (CAAS), Beijing, China; 2 Department of Agricultural Biotechnology, Biotechnology Center, Ethiopian Biotechnology Institute, Ministry of Innovation and Technology, Addis Ababa, Ethiopia; 3 Institute of animal science, Ningxia Academy of Agriculture and Forestry Sciences, Ningxia, Yinchuan, China; University of Florida, UNITED STATES

## Abstract

Coat colour is one of the most important economic traits of sheep and is mainly used for breed identification and characterization. This trait is determined by the biochemical function, availability and distribution of phaeomelanin and eumelanin pigments. In our study, we conducted a genome-wide association study to identify candidate genes and genetic variants associated with coat colour in 75 Chinese Tan sheep using the ovine 600K *SNP* BeadChip. Accordingly, we identified two significant SNPs (*rs*409651063 at 14.232 Mb and *rs*408511664 at 14.228 Mb) associated with coat colour in the *MC1R* gene on chromosome 14 with −log10(P) = 2.47E-14 and 1.00E-13, respectively. The consequence of rs409651063 was a missense variant (g.14231948 G>A) that caused an amino acid change (Asp105Asn); however, the second SNP (rs408511664) was a synonymous substitution and is an upstream variant (g.14228343G>A). Moreover, our PCR analysis revealed that the genotype of white sheep was exclusively homozygous (GG), whereas the genotypes of black-head sheep were mainly heterozygous (GA). Interestingly, allele-specific expression analysis (using the missense variant for the skin cDNA samples from black-head sheep) revealed that only the G allele was expressed in the skin covered with white hair, while both the G and A alleles were expressed in the skin covered with black hair. This finding indicated that the missense mutation that we identified is probably responsible for white coat colour in Tan sheep. Furthermore, qPCR analysis of *MC1R* mRNA level in the skin samples was significantly higher in black-head than white sheep and very significantly higher in GA than GG individuals. Taken together, these results help to elucidate the genetic mechanism underlying coat colour variation in Chinese indigenous sheep.

## Introduction

Animal coat colour is one of the most important economic traits, particularly for animal breeds that are kept for skin and wool production. Coat colour is a highly useful trait that affects the behaviour of animals and is an essential trait for survival (to cope with the environment and biological factors) in different environmental conditions [1–3]. Moreover, animal coat colour has played a crucial role in breed identification, characterization [4] and other applications, such as fibre production in goat, rabbit and sheep breeds [5]. In fact domesticated species are also characterized by a huge allelic variability of coat-colour-associated genes which leads to negative pleiotropic effects linked with coat-colour variants [6]. Animal coat colour variation is mainly regulated by genetic and environmental factors [6]. Classical genetics assumes that the presence of eumelanin or phaeomelanin is genetically controlled by the extension and agouti locus [7]. In mammals, coat colour variation is determined by the biochemical function, distribution and availability of phaeomelanin and eumelanin pigments [8]. Phaeomelanin produces a red and yellow colour, whereas eumelanin produces black and brown phenotypes [8]. The relative amount of eumelanin and phaeomelanin colours in melanocytes is regulated by the interactions of agouti signalling protein (*ASIP*) and melanocortin 1 receptor (*MC1R*) genes [9, 10]. In addition to *MC1R* and *ASIP*, there are also other genes involved in coat colour variation such as melanocyte inducing transcription factor (*MITF*), tyrosinase related protein-1 (*TYRP1*) and v-kit Hardy-Zuckerman 4 feline sarcoma viral oncogene homologue (*KIT*) [6, 11–15].

*ASIP* is a small paracrine signalling molecule that interacts with the extension locus encoded by the agouti locus [16]. *ASIP* has an antagonist function with *MC1R* in the pigmentation process. *ASIP* blocks the α-MSH receptor interaction, which causes pigment-type switching from eumelanin to phaeomelanin pigment [17, 18]. Thus, *ASIP* inhibits *MC1R* signalling and eumelanogenesis, and the antagonist function of *ASIP* promotes white and red colour against the black and brown phenotype [19–21]. The effect of *ASIP* on coat colour is determined by dominant or recessive agouti alleles. The dominant agouti alleles are responsible for phaeomelanin phenotypes and the recessive alleles for black coat colour [22]. Molecular variants have been reported in the *ASIP* gene associated with the coat colour phenotype in different sheep breeds, such as Finns sheep [23], Massese sheep [24], Soay sheep [25] and Australia merino sheep [4]. In contrast, the *MC1R* gene, also known as the α-melanocyte stimulating hormone receptor (α-MSHR), a seven-transmembrane G-protein coupled receptor, is encoded by an extension (E) locus [7]. The *MC1R* gene is reported as a potential candidate gene that plays a significant role in melanogenesis and wool pigmentation and is responsible for black coat colour in mammals [8, 26, 27]. For instance, several functional and non-functional molecular variants have been reported in the *MC1R* gene associated with coat colour phenotype in different sheep breeds such as Zandi, Baluchi and Zel sheep [28], Brazilian Crioula sheep [29], Manchega and Rasa Aragonesa sheep [30], Chinese sheep [15], Brazilian Creole sheep [8], Massese sheep [24], Xalda sheep [31], Norwegian Dala sheep [32].

**Fig 1:**
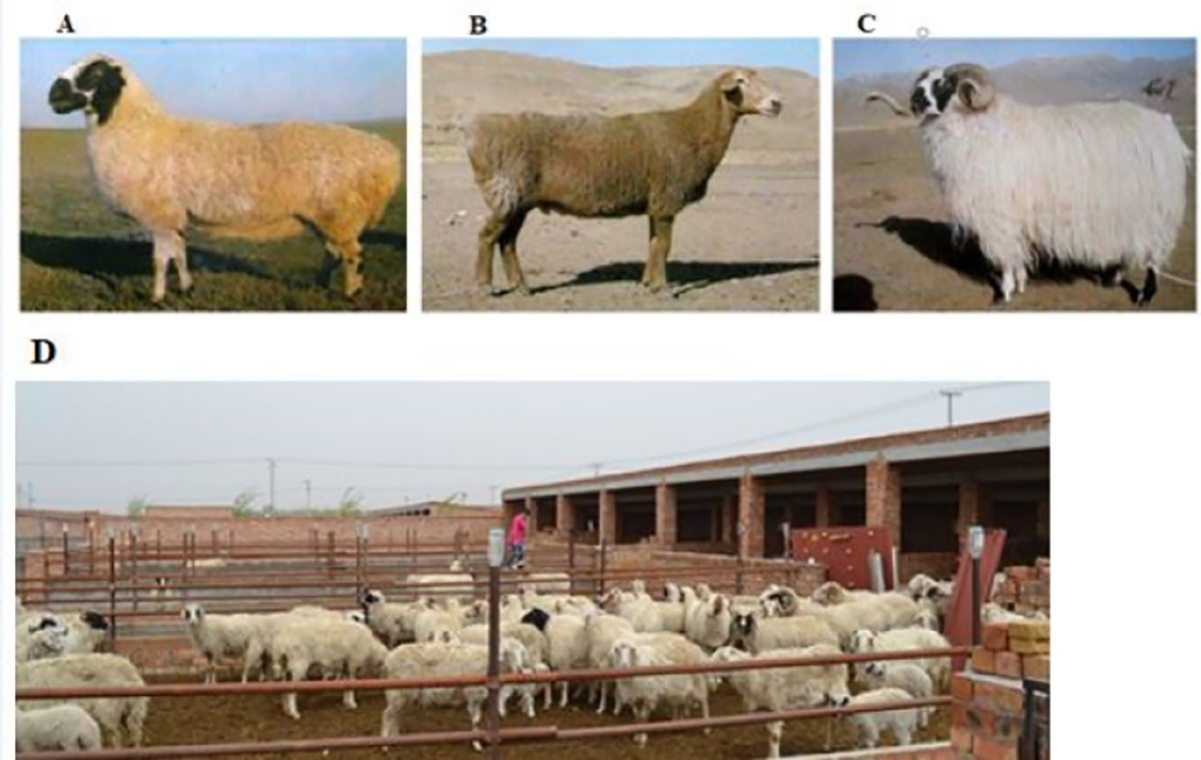
The typical image of the three categories of indigenous Chinese sheep breeds and Chinese Tan sheep. (A) Mongolian-fat tailed sheep group, (B) Kazakh-fat rumped sheep group, (C) Tibetan-thin-tailed sheep group and (D) shows solid white, white with black head and brown face phenotype of Tan sheep gathered in Ningxia conservation farm, Ningxia province, China.

Chinese indigenous sheep breeds are classified in 42 based on their geographical distribution and morphological characteristics. These sheep breeds are again categorized in three groups: Mongolian fat-tailed, Kazakh fat-rumped and Tibetan thin-tailed sheep (Fig 1A, 1B & 1C) [33]. Chinese Tan sheep are unique and typical sheep breeds that are employed for both fur and meat production and are categorized under the Mongolian fat-tailed sheep group. Tan sheep are widely distributed in northwestern China and famous for lamb pelt and lustrous white curly fleece production. These sheep are also renowned breeds for long-term adaptation to dry, cold and windy environments [34]. The coat colour of Chinese Tan sheep is solid white, and white with black and brown colours around the head, neck and face (Fig 1D). This coat colour variation among the Chinese Tan sheep was the main focus of our study. A genome-wide association study (GWAS) is a powerful and preferred approach to detect causal variants and define narrower genomic regions than Quantitative trait loci (QTLs). It is a hypothesis-free method to determine the associations between genetic regions and phenotypic traits in livestock and humans [35–37]. In sheep, GWAS was applied in Soay sheep for the first time to investigate horn types [35] and was subsequently applied to Corriedale sheep to examine the inheritance of rickets [36]. There are also GWAS reports for other traits in the Chinese sheep breed such as coat colour [13].

## Materials and methods

### Ethics approval

All the procedures involved in handling and collecting the samples from sheep were approved by the ethics committees of the Ministry of Agriculture of the People’s Republic of China (IASCAAS-AE-03).

### Animals and samples

**Fig 2:**
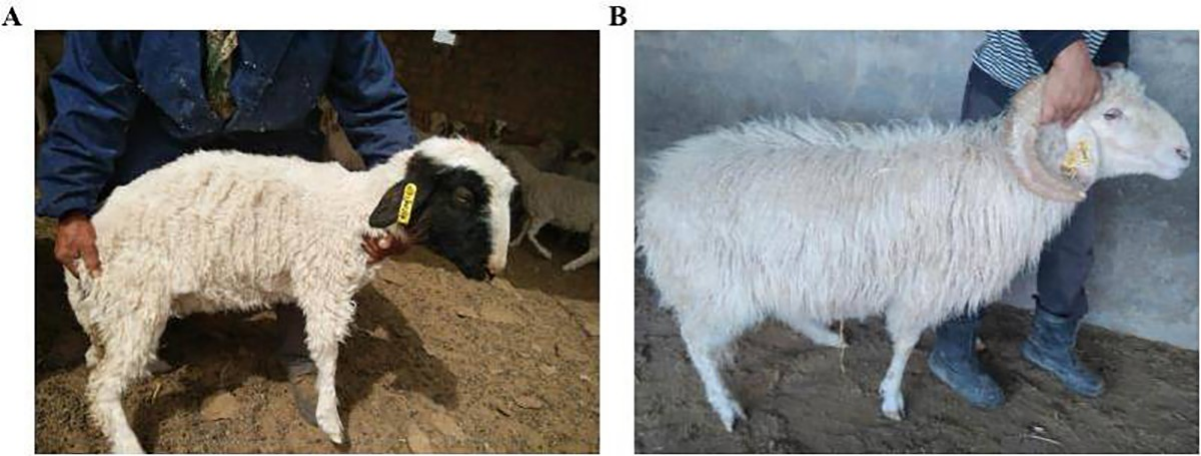
The coat colour phenotype of Chinese Tan sheep. (A) Shows white with black coat colour phenotype, whereas (B) referred to solid white coat colour phenotype.

For GWAS, a total of 10 ml of blood from the jugular vein into a tube and phenotype data were collected from 75 Chinese Tan sheep from the Ningxia sheep conservation farm, Ningxia province, China. Among the sampled sheep, 29 were coded as cases (black head/face coat colour), whereas 46 were coded as controls (white coat colour sheep) (Fig 2). For gene expression analysis, tissue samples (n = 14) were collected from the skin of solid white sheep and black-head sheep. Similarly, for allele-specific expression (ASE) analysis, tissue samples (n = 14) were collected from the skin under the black hair and under the white hair part of the black-head sheep (from the same sheep). The tissues were collected via skin punch biopsy under anaesthesia and placed in liquid nitrogen. Moreover, blood samples (n = 10) and tissue samples (n = 14) were collected from white and black-head Tan sheep to validate the gene.

### DNA and RNA extraction

Genomic DNA was extracted from 3-ml blood samples using Promega Wizard Genomic Purification according to the manufacturer’s protocol Kits, whereas RNA was extracted from skin tissue samples using the RNAprep Pure Kit (For Tissue). After DNA extraction, genotyping was performed using Illumina’s ovine 600K SNP HD BeadChip for GWAS work. For SNP identification, we performed polymerase chain reaction (PCR) using the reagent Phanta Max Super-Fidelity DNA Polymerase (Cat: P505-d1, Vazyme). Complementary DNA (cDNA) was also prepared from the extracted RNA samples using a PrimeScript^TM^ RT reagent kit for quantitative polymerase chain reaction (qPCR) and nested PCR.

### Genome wide association study (GWAS)

A case–control model was applied to manipulate the genotype data (S1 File and S2 File) and phenotype data (S3 File). Both genotype data quality control and phenotype-genotype association data generation were performed using PLINK version 1.9 software [38]. Manhattan plots and QQ plots were generated using the qqman package in R v.3.3.2. Genomic regions and candidate genes were identified on the livestock genome browser (UCSC) using the sheep assembly Aug. 2012 (ISGC Oar_v3.1/oviAri3). Finally, gene and SNP annotation was performed using Ensemble genome browser 95 and NCBI.

### Primer designing

Primers for qPCR, PCR and nested PCR were designed by Primer3Plus using the *MC1R* mRNA sequence *(*GenBank accession no. NM_001282528.1) and *GAPDH* mRNA sequences (GenBank accession no. NM_001190390.1). Glyceraldehyde-3-phosphate dehydrogenase (*GAPDH*) was used as a housekeeping gene for gene expression analysis. The primers were synthesized by Beijing Tianyihuiyuan Biotechnology Co. Ltd., Beijing-China (Table 1).

**Table 1:** Primer information for qPCR, nested PCR and PCR applications.

Primers name	Primer sequence	Product size	Applications	Locus/SNPs
*MC1R*-F *MC1R*-R	CTCTCCATCACCTACTACAACC CAGCATGTGGACATAGAGGAC	102 bp	For qPCR	14:14231948 (rs409651063)
*GAPDH*-F *GAPDH*-R	GTCCGTTGTGGATCTGACCT GGAGACAACCTGGTCCTCAG	130 bp		
*MC1R-*F1 *MC1R*-R1	GCTGGTGAGTCTTGTGGAGA GCCAAAGCCCTGATGAATGG	562 bp	For Nested PCR & PCR	14:14231948 (rs409651063)
*MC1R*-F2 *MC1R-*R2	GTGAGCGTCAGCAACGTG ACATAGAGGACGGCCATCAG	366 bp		
*MC1R*-F3 *MC1R*-R3	CCACCTGCTCTGCTCTTCTA GGAGGGTGCTCAGTAGACAA	466 bp	For PCR	14:14228343 (rs408511664)

Abbreviations: F, forward primer; R, reverse primer.

### Polymerase chain reaction (PCR)

PCR was performed in a total volume of 26 μL containing 2xPhanta Max Buffer (12.5 μL), dNTP Mix (0.5 μL), Phanta Max Super-Fidelity DNA Polymerase (0.5 μL), ddH_2_O (8.5 μL), forward primer (1 μL), reverse primer (1 μL) and 2 μL of DNA. The PCR analysis was performed on an Applied Biosystems® Gene Amp® PCR System 9700 and programmed as follows: at 95°C initial denaturation for 5 min, 35 amplification cycles of 30 s denaturing at 95°C, 30 s annealing at 54°C and extension at 72°C for 2 min. The final extension was run at 72°C for 5 min. Agarose gel electrophoresis was carried out to determine whether the PCR products properly amplified the target region using 1.5% agarose, 50 ml 1X TAE buffer and 5 μL God view. The gel was checked using a molecular imager (Gel Doc XR, BIO-RAD). Care was taken to avoid DNA damage due to extended exposure to UV light.

### cDNA preparation

The Complementary DNA (cDNA) was synthesized from 1 μg of DNase-treated RNA using a primeScript^TM^ RT reagent kit. First, we prepared a total volume of 10 μL of DNA elimination reaction containing 5× gDNA Eraser Buffer (2 μL), gDNA Eraser (1 μL), RNA (1 μL) and RNase Free ddH_2_O (6 μL). Next, 10 μL of master mix was composed of 5X PrimeScript Buffer 2 (4 μL), PrimerScript RT Enzyme Mix I (1 μL), RT primer Mix (1 μL) and RNase Free ddH_2_O (4 μL). Then, the DNA elimination reaction and master mix were mixed to prepare 20 μL of reverse transcription reaction. Finally, the cDNA solution was heated on a Veriti 96-well thermal cycler at 37°C for 15 min and at 85°C for 5 s. This solution was diluted with ddH_2_O and stored at 4°C for PCR, nested PCR and qPCR use.

qPCR was performed in a total volume of 20 μL containing 10 μL of TB Green Premix Ex Taq, 0.4 μL of forward primer, 0.4 μL of reverse primer, 0.4 μL of ROX Reference Dye II, 2 μL of diluted cDNA and 6.8 μL of ddH_2_O. The cycling parameters were 95°C for 30 s followed by 40 cycles (95°C for 5 s, 60°C for 34 s and 95°C for 15 s) and a cycle (60°C for 1 min and 95°C for 15 s). Nested PCR was also prepared using two sets of primers (562 bp and 366 bp) (Table 1). The first PCR was prepared using the first set of primers in a total volume of 20 μL composed of 1 μL of forward primer, 1 μL of reverse primer, 10 μL of Master mix, 6 μL of ddH_2_O and 2 μL of cDNA solution. The cycling parameters were 95°C for 5 min, 34 cycles (95°C for 30 s, 60°C for 30 s and 72°C for 1 min) and 72°C for 2 min and held at 4°C. The second reaction was performed using the second set of primers and 1 μL of PCR product from the first PCR (instead of cDNA template). All other reaction components and cycling parameters remained the same as those of the first reaction. Agarose gel electrophoresis was carried out using 1.5% agarose, 50 ml 1X TAE buffer and 5 μL God view to amplify PCR products.

### DNA genotyping and gene expression analysis

The gene (*MC1R*) and the genetic variants (*rs*409651063 and rs408511664) identified to be associated with the coat colour phenotype were confirmed using PCR approaches. For this, PCR products (from DNA and cDNA) were sequenced by Beijing Liuhe BGI Technology Co., Ltd. The DNA and cDNA sequence results were assembled, aligned and edited using MEGA 7.0.14 (7160202-x86_64) to identify the genetic variants and for allele-specific expression analysis. SNP positions were cross-checked and verified by Chromas version 2.6.4 software. Moreover, qPCR data were analysed using GraphPad Prism v6.04 to determine whether the *MC1R* gene is differentially expressed in white and black-head Chinese Tan sheep.

## Results and discussion

### Results

**Fig 3:**
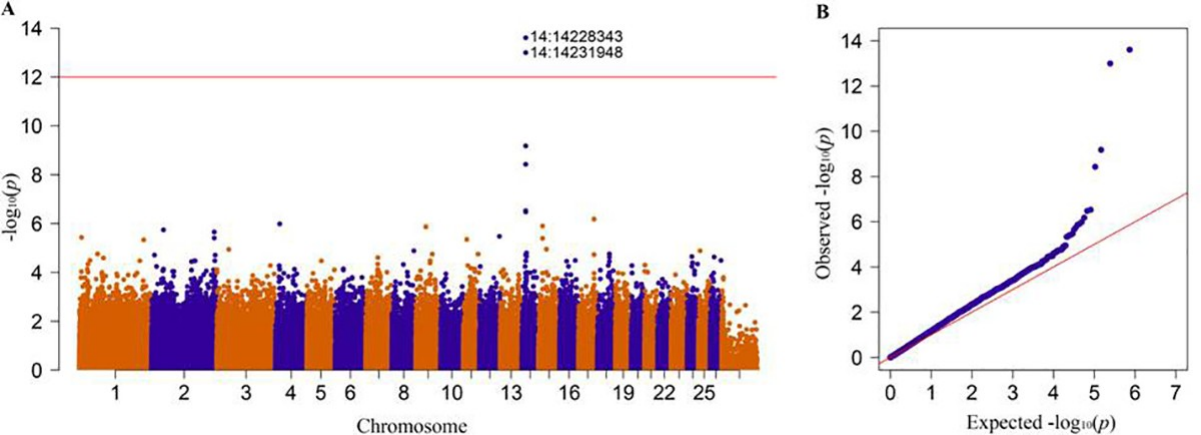
GWAS results plotted using Manhattan and quantile-quantile plots. (A) Manhattan plot for coat colour analysis. The horizontal red line denotes genome-wide significance (1e^-12)^. The y-axis shows the -log_10_ (*p*) value, and the x-axis shows the chromosome number. (B) QQ plot: the -log10 (P-value) shows the normality of the observed (y-axis) and expected (x-axis) values. The red line shows a normal curve, -and blue indicates colour variation among individuals. The two blue dots on the top denote significant SNPs for the coat colour phenotype.

GWAS: after quality control was performed using missing genotype data (0.1), Hardy-Weinberg equilibrium (1e-5) and minor allele frequency (0.05), a total of 366,991 SNPs remained for the genome-wide association study. Then, we perform the GWAS using phenotype data and found a candidate genomic region spanning 14.228Mb-14.232Mb on chromosome 14. The genomic location (SNPs) was then ranked using their p-value (smallest to largest) (Table 2). The Manhattan plot result indicated the significant SNPs as top signals for the black-head phenotype at chromosome 14 (Fig 3A). Moreover, the quantile-quantile plot (QQ plot) was also examined to determine the validity of the P-value of the significant SNPs. The corresponding QQ plot showed a reasonable distribution of the SNPs on GWAS. Most of the SNPs followed the normal curve distribution; however, only a few significant SNPs deviated from the normal curve for the black-head coat colour phenotype (Fig 3B).

**Table 2:** GWAS results: genetic variants and their location, *P*-value, amino acid change and consequences.

SNPs	Chr:bp	Alleles	P-value	Consequence Type	AA	Gene
rs408511664	14:14228343	G/A	2.47E-14	upstream gene variant		*MC1R*
rs409651063	14:14231948	G/A	1.00E-13	missense variant	D/N	
rs160909987	14:14227752	C/T	6.62E-10	upstream variant		*MC1R*
rs409417796	14:14213367	G/A	3.74E-09	intron variant		*MC1R*
rs412176113	14:14216978	C/T	2.96E-07	intron variant		*MC1R*

Abbreviations: SNP, single nucleotide polymorphism; Chr, chromosome; BP, base pair position; AA, amino acid change.

We were also identified two significant SNPs (*rs*409651063 at 14.232 Mb position and *rs*408511664 at 14.228 Mb position) on chromosome 14, with −log10(P) = 2.47E-14 and 1.00E-13, respectively. These SNPs were identified on coding (CDS) and upstream regions of the *MC1R* gene. The consequence of the variant rs409651063 was a missense variant (g.14231948 G>A) causing an amino acid change (Asp105Asn); however, the second SNP (rs408511664) was an upstream variant (g.14228343G>A) (Table 2). The SIFT prediction value for the rs409651063 variant was smaller than 0.05 (SIFT score = 0.01). The effect of this genetic variant on *MC1R* protein function is deleterious. This finding suggested that *MC1R* signalling may be disrupted by the mutation.

### Population genetic structure analyses

**Fig 4:**
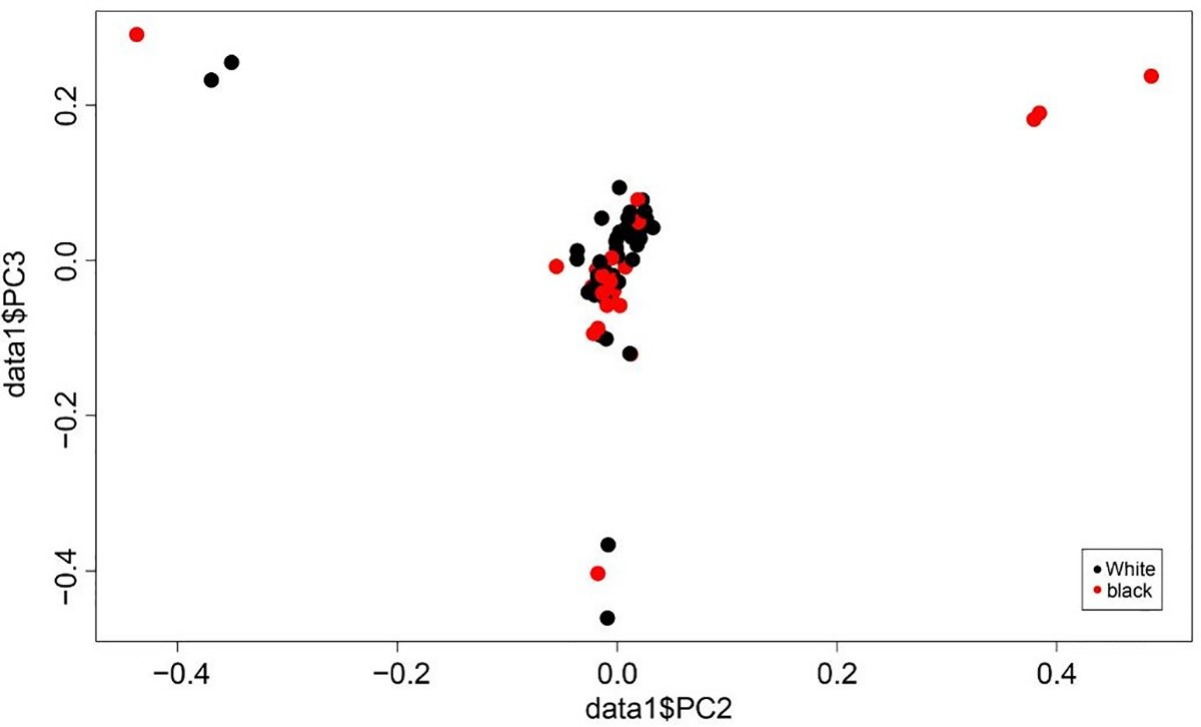
Principal component analyses of white and black-head Tan sheep.

Population structure was performed to characterize the genetic relationship between the animals with two types of coat colour phenotypes (white Tan sheep and black-head Tan sheep) using principal component analysis (PCA). Accordingly, we observed a closer clustering pattern between the phenotypes implied that the animals are closely related (Fig 4).

### Validation of the GWAS results

After sequencing the DNA by Sanger sequencing, DNA genotyping revealed that all the individuals from the white phenotype were homozygous (GG), whereas all individuals from the black-head phenotype were heterozygous (GA) (DNA genotype, Table 3). Moreover, we performed allele-specific expression (ASE) analysis for the missense variant (*rs*409651063) to investigate the effects of the mutation in the skin samples covered with either white hair or black hair. The combined results of the DNA genotype and cDNA genotype of the missense variant (rs409651063) revealed that only allele G was expressed in the skin covered with white hair, while both G and A alleles were expressed in the skin covered with black hair (Table 3). This preference of the mutant G allele in the skin covered with white hair indicated that the resulting mutant *MC1R* protein may lead to dysfunction of black pigmentation in white skin.

**Table 3:** DNA genotyping and allele-specific expression (ASE) analysis for two candidate loci: chr14:14228343 (rs408511664) and chr14:14231948 (rs409651063).

blood sample	coat colour phenotype	DNA genotype		Skin sample	hair colour of skin	cDNA genotype (14231948)
		14228343	14231948			
B1	black head	GA	GA	B1-1	black	GA
				B1-2	white	GG
B2	black head	GA	GA	B2-1	black	GA
				B2-2	white	GA
B3	black head	GA	GA	B3-1	black	GA
				B3-2	white	GG
B4	black head	GA	GA	B4-1	black	GA
				B4-2	white	GA
W1	white	GG	GG	W1	white	GG
W2	white	GG	GG	W2	white	GG
W3	white	GG	GG	W3	white	GG
W4	white	GG	GG	W4	white	GG
W5	white	GG	GG	W5	white	GG
W6	white	GG	GG	W6	white	GG

Abbreviations: B1, black head sheep sample 1; B1-1, black colour skin from sample 1; B1-2, white colour skin from sample 1; B2, black head sheep sample 2; B2-1, black colour skin from sample 2; B2-2, white colour skin from sample 2, the same way of coding to other samples also.

**Fig 5:**
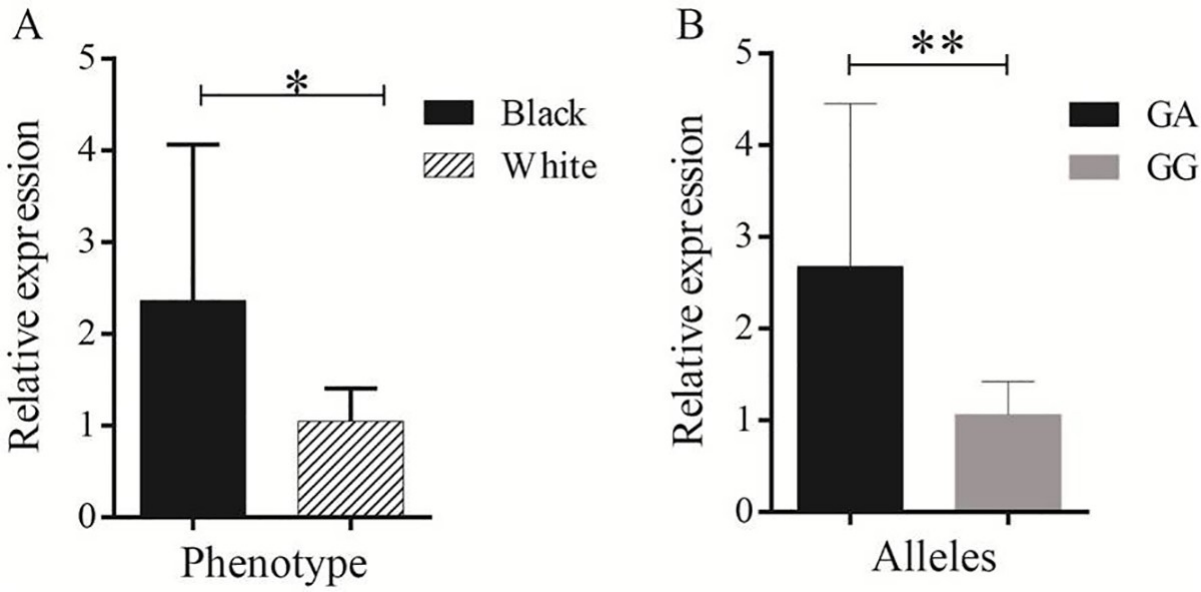
Gene and allele expression level for white and black coat colours phenotype of Chinese Tan sheep. (A) Comparison of gene expression (*MC1R)* in black-head and white colour sheep. (B) Expression level comparison between the GG genotype (white coat colour sheep) and GA genotype (black-head coat colour sheep) in the *MC1R* gene for the rs409651063 variant. The sign * is denoted for *P* < 0.05 and ** is for *P* <0.01.

Furthermore, we performed qPCR for the missense variant (rs409651063) to evaluate the expression level of the *MC1R* gene in white and black-head phenotypes. Accordingly, *MC1R* mRNA was differentially expressed in the white and black-head phenotypes in Tan sheep. The expression level was significantly higher in sheep with black-head coat colour carrying the A allele than in white sheep (Fig 5A). The *MC1R* mRNA expression level in the skin samples was also significantly higher in the individuals carrying GA alleles than in the individuals carrying GG alleles (Fig 5B). The gene expression cycle threshold (Ct) value of *MC1R* and *GADPH* (reference) genes including replicates of each sample is available in the supplementary file (S1 Table).

## Discussion

Chinese Tan sheep (widely distributed in northwestern China) are unique and typical sheep breed that are reared for both fur and meat production. These sheep are renowned for long-term adaptation to dry, cold and windy environments. The coat colour of this breed is characterized by sold white and white colours with black and brown colours around the head, neck and face [33]. Thus, it would be important to identify the genomic regions and genetic variants associated with coat colour traits in Tan sheep.

In the present GWAS study, we identified a candidate gene (*MC1R*) and two significant genetic variants (*rs*409651063 and *rs*408511664) associated with coat colour in Tan sheep. This gene and the molecular variants were detected in the genomic region spanning 14.231 Mb-14.228 Mb on chromosome 14 (Table 2 and Fig 3A and 3B). According to Kijas et al. [30], a GWAS (SNP50 BeadChip) identified two SNPs (s26449 and s46705) closest to the *MC1R* gene on chromosome 14 in solid black and white Manchega and Rasa Aragonesa sheep associated with the coat colour phenotype. The variants were located 14.127 Mb upstream and 14.203 Mb downstream of the *MC1R* gene. However, the SNP density applied and the phenotype of sampled animals were not similar to those in our study. In a previous PCR study, three synonymous and two nonsynonymous variants of the *MC1R* gene were reported in completely black Chinese sheep (Minxian Black-fur breed). These variants of the *MC1R* gene were found to be associated with the black coat colour phenotype in the Minxian black-fur breed, although no complete association was reported in the black-face coat colour phenotype of Chinese sheep breeds (Tan, Tibetan, Duoland and Mongolian sheep) [15]. Moreover, Yang et al.[15] reported three haplotypes in the *MC1R* gene related to the black coat colour phenotype in Minxian black-fur sheep. In fact, in the present study, we identified a missense variant (g.14231948 G>A) causing p.Asp105Asn amino acid change and an upstream variant (g.14228343 G>A) (Table 2) in Chinese Tan sheep. This finding indicated that pigmentation variation in Chinese Tan sheep might be due to these genetic variants occurring on the CDS and upstream region of the *MC1R* gene. Fontanesi et al. [39] also reported missense and nonsense mutations in the *MC1R* gene in goat breeds. Moreover, several molecular studies, including GWAS, have been conducted to investigate the genetic variation in coat colour phenotype in sheep breeds. In such studies, the *MC1R* gene is labelled to be responsible for black colour phenotypes in sheep [5, 30, 40]. Muniz et al. [40] reported the seven most significant SNPs on chromosome 14 close to the *MC1R* gene in Morada Nova sheep in his GWAS study to investigate genome regions responsible for the coat colour phenotype. Furthermore, several SNPs have been observed in the *MC1R* gene in different animal species associated with the coat colour phenotype. For instance, in a recent study, two silent mutations in the 5' end of the coding region of the *MC1R* gene were detected in Iranian sheep breeds with synonymous effects on amino acid sequences [28]. However, generally, the *MC1R* gene is not the only genetic factor that determines the coat colour phenotype; there are also genes, such as *ASIP*, that are responsible for shaping the coat colour of sheep and other animal species [4, 5, 20, 23–25, 31].

We took one step further to validate the two variants identified in our GWAS analysis. We confirmed the association of deleterious substitution (rs409651063) and the upstream variant (rs408511664) with coat colour phenotype in an extra population using Sanger sequencing (DNA genotype, Table 3). Moreover, in our allele-specific expression analysis of the missense variant, we found that the mutant G allele was likely to be expressed in the skin of white hair from black head sheep, whereas the black part tended to express the reference A allele (cDNA genotype, Table 3). These results suggested that the missense mutation probably disrupted the black pigmentation receptor of *MC1R* and thus prevented normal pigmentation of the hair. The upstream variant (*rs*408511664) was also found to be associated with coat colour phenotype which might have regulatory effects of this mutation. Furthermore, the expression level of *MC1R* mRNA was significantly higher in sheep with black-head coat colour carrying the A allele than in white sheep (Fig 5A). Similarly, the *MC1R* mRNA expression was significantly higher in the individuals carrying GA alleles than in the individuals carrying GG alleles (Fig 5B). This finding showed that the *MC1R* gene has a great role in black-colour pigmentation process. The novel discovery of the two variants and allelic expression imbalance of the *MC1R* mutant and reference alleles helped to elucidate the mechanism of *MC1R* transcription and the genetic mechanism underlying coat colour in sheep.

In conclusion, we identified a genomic region, a candidate gene and genetic variants associated with the coat colour phenotype in Chinese Tan sheep using GWAS and the Ovine 600K SNP BeadChip. The genetic variants in *MC1R* may affect the coat colour phenotype of Chinese Tan sheep; however, other genes might have their own role in the pigmentation-making process. Moreover, the *MC1R* gene expression level was significantly higher in black-head Tan sheep than in white sheep and significantly higher in GA alleles than in GG allele individuals. Thus, *MC1R* may have a significant role in regulating melanin synthesis and the development of black skin in Chinese Tan sheep. Furthermore, allele G was expressed in the skin covered with white hair, whereas both G and A alleles were expressed in the skin covered with black hair. Further studies are needed to explore other candidate genes, such as *ASIP*, and to determine their biological effect on coat colour formation in Tan sheep.

## Supporting information

S1 File This is the S1 map data file used for GWAS in this study. (MAP)

S2 File This is the S2 ped data file used for GWAS in this study. (PED)

S3 File This is the S3 phenotype data file used for GWAS in this study. (TXT)

S1 Table This is the S1 gene expression data file used for gene expression analysis. (DOCX)
