# Correction: Genomic mapping identifies two genetic variants in the *MC1R* gene for coat colour variation in Chinese Tan sheep

**DOI:** 10.1371/journal.pone.0245674

**Published:** 2021-01-13

**Authors:** Gebremedhin Gebreselassie, Benmeng Liang, Haile Berihulay, Rabiul Islam, Adam Abied, Lin Jiang, Zhengwei Zhao, Yuehui Ma

The fourth author's name is spelled incorrectly. The correct name is: Rabiul Islam. The correct citation is: Gebreselassie G, Liang B, Berihulay H, Islam R, Abied A, Jiang L, et al. (2020) Genomic mapping identifies two genetic variants in the *MC1R* gene for coat colour variation in Chinese Tan sheep. PLoS ONE 15(8): e0235426. https://doi.org/10.1371/journal.pone.0235426
