# Supplementary material for: Genomic mapping identifies two genetic variants in the MC1R gene for coat colour variation in Chinese Tan sheep
Source: PLoS One. 2020 Aug 20;15(8):e0235426. doi: 10.1371/journal.pone.0235426 (PMC7444486; doi:10.1371/journal.pone.0235426)
Supplement: S1 Table — (DOCX) [file pone.0235426.s004.docx]

| **Sample Name** | **MC1R(ct）** | **Sample Name** | **GAPDH（ct)** |
| --- | --- | --- | --- |
| B1-1 | 27.944 | B1-1 | 20.658 |
| B1-1 | 28.109 | B1-1 | 20.626 |
| B1-1 | 27.739 | B1-1 | 20.359 |
| B2-1 | 29.523 | B2-1 | 20.360 |
| B2-1 | 29.871 | B2-1 | 20.166 |
| B2-1 | 30.102 | B2-1 | 20.357 |
| B3-1 | 28.524 | B3-1 | 19.049 |
| B3-1 | 28.409 | B3-1 | 18.988 |
| B3-1 | 28.590 | B3-1 | 18.992 |
| B3-2 | 28.997 | B3-2 | 19.404 |
| B3-2 | 29.116 | B3-2 | 19.420 |
| B3-2 | 29.076 | B3-2 | 19.453 |
| B4-1 | 29.034 | B4-1 | 20.798 |
| B4-1 | 29.290 | B4-1 | 20.925 |
| B4-1 | 29.076 | B4-1 | 20.936 |
| W1 | 30.439 | W1 | 21.669 |
| W1 | 31.078 | W1 | 21.570 |
| W1 | 31.194 | W1 | 21.509 |
| W2 | 29.287 | W2 | 19.642 |
| W2 | 29.355 | W2 | 19.650 |
| W2 | 29.368 | W2 | 19.599 |
| W3 | 30.075 | W3 | 19.910 |
| W3 | 29.597 | W3 | 19.989 |
| W3 | 30.244 | W3 | 19.985 |
| W4 | 29.037 | W4 | 19.139 |
| W4 | 29.269 | W4 | 19.209 |
| W4 | 29.572 | W4 | 19.095 |

|  | **Table S1 a table with the expression results (Ct) of MC1R and GADPH genes including replicates of each sample.** |
| --- | --- |
